# E-Rehabilitation – an Internet and mobile phone based tailored intervention to enhance self-management of Cardiovascular Disease: study protocol for a randomized controlled trial

**DOI:** 10.1186/1471-2261-12-50

**Published:** 2012-07-09

**Authors:** Konstantinos Antypas, Silje C Wangberg

**Affiliations:** 1 Norwegian Centre for Integrated Care and Telemedicine, University Hospital of North Norway, P.O. Box 35, Tromsø, N-9038, Norway; 2 Regional Centre for Substance Use, University Hospital of North Norway, P.O. Box 385, Narvik, N-8505, Norway; 3 Department of Clinical Medicine, Faculty of Health Sciences, University of Tromsø, Tromsø, N-9037, Norway

**Keywords:** Tailoring, Cardiac rehabilitation, Cardiovascular disease, EHealth, Internet-based, Mobile-based, Self-management, Physical activity

## Abstract

**Background:**

Cardiac rehabilitation is very important for the recovery and the secondary prevention of cardiovascular disease, and one of its main strategies is to increase the level of physical activity. Internet and mobile phone based interventions have been successfully used to help people to achieve this. One of the components that are related to the efficacy of these interventions is tailoring of content to the individual. This trial is studying the effect of a longitudinally tailored Internet and mobile phone based intervention that is based on models of health behaviour, on the level of physical activity and the adherence to the intervention, as an extension of a face-to-face cardiac rehabilitation stay.

**Methods/Design:**

A parallel group, cluster randomized controlled trial. The study population is adult participants of a cardiac rehabilitation programme in Norway with home Internet access and mobile phone, who in monthly clusters are randomized to the control or the intervention condition. Participants have access to a website with information regarding cardiac rehabilitation, an online discussion forum and an online activity calendar. Those randomized to the intervention condition, receive in addition tailored content based on models of health behaviour, through the website and mobile text messages. The objective is to assess the effect of the intervention on maintenance of self-management behaviours after the rehabilitation stay. Main outcome is the level of physical activity one month, three months and one year after the end of the cardiac rehabilitation programme. The randomization of clusters is based on a true random number online service, and participants, investigators and outcome assessor are blinded to the condition of the clusters.

**Discussion:**

The study suggests a theory-based intervention that combines models of health behaviour in an innovative way, in order to tailor the delivered content. The users have been actively involved in its design, and because of the use of Open-Source software, the intervention can easily and at low-cost be reproduced and expanded by others. Challenges are the recruitment in the elderly population and the possible underrepresentation of women in the study sample. Funding by Northern Norway Regional Health Authority.

**Trial registration:**

Trial registry http://www.clinicaltrials.gov: NCT01223170.

## Background

Cardiovascular diseases (CVD) according to the World Health Organization (WHO) are leading causes of death and represent 30% of all global deaths and 48% of the deaths in Europe [1,2]. Moreover, the current trends predict increase in deaths caused by CVD over the next years. Secondary preventive efforts can decrease mortality risk as well as increase health among CVD patients. This study suggests a new approach in supporting the self-management of CVD patients after rehabilitation. The experimental research model that is implied investigates the effectiveness of tailoring an Internet- and mobile-based intervention according to concepts derived from models of health behaviour, and will contribute to develop these further. If successful, concepts from this project can potentially be extended to primary prevention of CVD among high-risk groups.

The prevention policy for CVD is based on strong scientific evidence and is focusing on three major health behaviours: smoking cessation, healthy diet, and increase of physical activity [3-5]. A rehabilitation and secondary preventive strategy based on the adoption of the same health behaviours after the diagnosis and/or symptoms can decrease mortality risk and increase quality of life [4,5]. Involvement in physical activity is a lifestyle modification that is crucial for the post-discharge period. Exercise interventions can cause a 27% reduction in total mortality and a 31% in cardiac mortality in patients with Coronary Heart Disease [6]. Post-discharge physical activity programmes can take place in hospital setting or at home with no significant differences in physiological and psychological outcomes [7]. The achieved lifestyle changes, however, often prove difficult to maintain [8].

Social support might be one way of increasing long-term adherence to exercise. One likely mechanism through which peer support is important in lifestyle change, is by providing modelling of overcoming barriers towards successful behaviour, thus increasing the belief of the individual that he can succeed in changing his own behaviour (self-efficacy) [9]. However, not everyone has the opportunity to participate in face-to-face groups, so the Internet might provide a good alternative *e.g.*[10].

Internet-based interventions have a large potential for reaching people. In 2008, 67% of Norwegians reported having used the Internet for health purposes [11]. In general, reviews of Internet-based interventions might present a rather confusing picture, as the only common ground is the delivery medium. The interventions include online publish of pamphlets, combinations of text-based information and communicative features such as forums, “ask an expert” services, individually computer tailored content, and multimedia iterations. Nevertheless, a review of six Internet-based interventions for supporting diabetes self-management found that five of the six studies reported improvements in health behaviours [12]. A more general review of Interactive Health Communication Applications (IHCA) for people with chronic diseases concluded that these applications improved users’ knowledge, social support, health behaviours and clinical outcomes [13].

Many of the most successful Internet-based interventions for supporting change in health behaviours utilise tailored content. A tailored intervention is an intervention that is adapted to the characteristics of the individual, typically based on responses to a questionnaire [14]. Tailored interventions have generally proven more effective than standardized self-help materials for smoking cessation [15,16]. Previous research has shown that tailored health messages are in general perceived as more interesting and personally relevant, are better liked, read more thoroughly, discussed more, and remembered better compared to non-tailored educational material [17-20].

Although the tailoring component in Internet-based interventions has received some attention, to the best of our knowledge, no studies have yet compared two versions of an Internet-based intervention for supporting CVD self-management with and without tailoring. A system that additionally can monitor physical activity can give feedback to the patient about the efficacy of the exercise, indicating the exercise capacity of the patient [21]. A review of eHealth interventions for increasing physical activity indicated that these could increase the number of steps, walking minutes and level of activity. Physiological indicators validated the indicated increase of physical activity, *i.e.* increase of heart rate and VO_2_ max and decrease of percentage of body fat [22]. From the same review it is suggested that higher utilization and dose of intervention have better results in health behaviour change, that peer support modules increase log-on rates and that most users read the information they received electronically.

In addition to the bibliographic evidence, we conducted a focus group with 11 CVD patients of the Rehabilitation Centre where the study is going to take place. During a semi-structured discussion with them, the potential users of our service expressed the need for continuing support after they are discharged from the rehabilitation centre. They said that, when they go back home, they need help to remain active, to remain in contact with other participants they met at the rehabilitation centre, to set realistic goals, to plan according to those goals and to receive feedback on their effort to remain physically active. One participant suggested: “What if we could get an email every day at a specific time? Time for exercise!” and when the discussion was about the usability of an exercise diary another participant said: “…a diary where you can show others what you have done…maybe it could create some sort of competitive drive between some of the users. It could make it feel more like an obligation”. Generally participants were positive to the idea of a system that could support them in extending the positive impact the rehabilitation programme had on them. To meet that need, we have to find a way to bring the intervention next to the participants in the same way their peers and the personnel is at the Rehabilitation Centre. Mobile technology seems to be the most appropriate tool since it can be portable and be carried by the participant everywhere.

### Aims and hypotheses

Our aim is to assess the effects of a tailored Internet- and mobile-based intervention on maintenance of self-management behaviours after a cardiac rehabilitation stay. Our hypothesis is that the intervention group (tailored) will have higher adherence to the Internet-based intervention, and be more physically active.

## Methods

### Tailoring

Models of health behaviour often form the core of adaptive tailored interventions. It is therefore important to have adequate process measures in these kinds of interventions in order to identify which variables one should tailor to, for whom, and when. Self-efficacy is one of the theoretical constructs thus far having shown the most consistent effects of being tailored to [23]. Studies have indicated that the stage of change for the cardiac rehabilitation patient when entering an exercise programme is related to outcome, and is therefore another relevant variable to tailor interventions to [24]. We propose to combine and extend this research in line with the Health Action Process Approach HAPA [25], through tailoring to different self-efficacies according to where the individual are in their process of change. A third variable that we will tailor on is the individuals’ promotion- or prevention-goal orientation (regulatory focus). Latimer and colleagues have shown that tailoring to regulatory focus can increase both physical activity [26] as well as fruit and vegetable intake [27].

### Study population

#### Participants

The population of the study is recruited among the participants of the Skibotn Rehabilitation Centre’s cardiac rehabilitation programme. Participants to that programme are people with cardiovascular diseases that usually have been discharged from the region’s main hospital, the University Hospital of North Norway, during a period of 6 months before the programme’s start. They are referred to the Centre by their family doctor and a recent cardiac stress test is required. The cost of the four-weeks programme is partially covered by the participants and the Norwegian Health Scheme covers the rest.

#### Inclusion criteria

The study is open to all the participants of the cardiac rehabilitation programme of the Skibotn Rehabilitation Centre that have access to Internet after their stay at Skibotn, have a personal mobile phone, are willing to participate and have signed the informed consent form.

#### Sample size estimation

Power analysis for *a priori* sample size estimation was performed with equivalence test for two-proportions in a cluster-randomized design by the computer program PASS [28]. The participants that attend the cardiac rehabilitation programme together a given month, consist a cluster. Based on previous research with chronic disease self-management [29] it is reasonable to expect that the proportions meeting goals for all self-management behaviours at one-year follow-up will be relatively low *e.g.*, 15% *vs.* 5%. To discover differences of this size between two groups at a 0.05 alpha level and with 0.80 power, we need a total sample of 16 clusters with 15 participants each. We will recruit a total of 17 clusters, equalling continuous recruitment of all the participants of the cardiac rehabilitation programme of Skibotn Centre over 18 months.

### Ethical aspects

The study protocol, its updates and all the questionnaires have been submitted for approval by the Regional Ethics Committee. The study uses a convenience sample, *i.e.*, the participants have volunteered. Participation in the study is based on informed consent. In addition, an extensive Risk Assessment Report has been produced to identify potential risks and appropriate practices have been adopted to minimize the identified risks. For example all data will be gathered and stored in de-identified form and user-related data will be secured through necessary encryption and authentication.

## Design

Skibotn Rehabilitation Centre receives each month (except from July) a group of 10–20 CVD patients that are admitted in the rehabilitation programme. Since these people are hosted in the same environment for all of this period and social interaction related to the rehabilitation is encouraged, a randomisation among the members of the same group would make it very likely for patients to realize whether they belong to the control or intervention branch. In order to minimize this risk of contamination of the control group, the participants will be blinded to condition by randomizing the monthly clusters of participants to one of two conditions:

(1) A control condition involving a basic Internet-based self-management intervention

(2) An experimental condition where the participants get access to a tailored and enhanced version of the Internet-based intervention

The researchers are also blinded for the condition of each monthly group, so we will avoid introducing any bias in the analysis of the data. To ensure true randomness of the groups, we used random.org to allocate the groups to the two conditions. Random.org is a true random number online service that is based on the atmospheric noise. The duration of the study is one year after the discharge from the cardiac rehabilitation programme.

### Control internet-based intervention

All participants will be given access to a basic Internet-based intervention, “ikkegideg.no” (Norwegian for “Don’t give up”), consisting of general information about CVD and self-management, including diet, physical activity, smoking and medication, and a discussion forum. In the discussion forum we have two levels of accessibility. There is the closed groups level, where the users are able to create and take part in discussions that are only accessible by those that are members in the same monthly group. Users that belong to the same monthly group have met physically during the rehabilitation programme and it is expected that the level of trust among them will be higher. At the same time, all the users will be able to create, read and take part in discussions that will be visible by all the registered users of the website. This helps the discussion forum to become more active, since more users will be contributing to the discussions.

### Enhanced internet-based intervention

In the intervention group, the participants will in addition to general information and the discussion forum have access to the following: (1) content tailored to stage of change, regulatory focus and self-efficacy; (2) behavioural monitoring (self-reported physical activity).

The tailoring process is in some ways emulating face-to-face patient counselling. The answers that patient gives to the questionnaires creates an individualized path through the intervention, including feedback and follow-up questions based on predefined algorithms. Different answers to the same questions over the time generate changes in the treatment or behaviour change plan that reflect the changes in a patient’s characteristics or change process [14]. The adaptive tailoring in this intervention is based on integrative models that combine socio-cognitive determinants of health behaviour with a process view, such as the Integrated Model for explaining motivational and behavioural change I-Change [30] and the Health Action Process Approach HAPA [25]. Thus, we tailor to stage of change [24], which determine when the other concepts are tailored on; for instance, self-efficacies [25,29,31], and regulatory focus [26,32].

### Procedure

During the first days of the stay of each monthly group at the Centre, a physiotherapist, who is member of the staff there and administrator of the site, presents the website and the study, answers questions and distributes the consent form to the participants that are interested. Then another session is scheduled for those participants that decide to sign the consent form and take part in the study. In this additional session, the participants are registering to the website, fill-in online the baseline questionnaire and they receive training on how to use the website. During these two meetings the potential participants are generally informed of the features of the site and of the two study conditions, but not in such detail that it will be possible for them to understand in which group they belong.

At the end of the rehabilitation stay, participants are offered a repetition Internet-training session, and are being asked to log-on to fill out the Post-rehab questionnaire. The participants are being encouraged to keep visiting the intervention website after they return home, and are given prompts by email and SMS (Short message service) to fill out follow-up questionnaires at 1, 3, and 12 months after leaving the centre.

### Measures

For an overview of all measures over the different measurement time points, please see Table 1.

**Table 1:** Overview of measures over the different time points of the study

	**Baseline**	**Post-rehab**	**1 month**	**3 months**	**12 months**
Socio-economic status	Y				
Co-morbidity	Y				Y
Alcohol use	Y				Y
Health-related Internet use	Y				Y
Disease related symptoms	Y	Y	Y	Y	Y
iPAQ	Y	Y	Y	Y	Y
Social support	Y	Y	Y	Y	Y
Self-efficacies	Y	Y	Y	Y	Y
HADS	Y	Y	Y	Y	Y
Perceived Tailoring			Y	Y	Y
Stage of change	Y	Y	Y	Y	Y
Decisional balance	Y	Y	Y	Y	Y
Regulatory focus	Y				
7-day smoking abstinence	Y	Y	Y	Y	Y
Quality of life	Y	Y	Y	Y	Y
Usage logging	Y	Y	Y	Y	Y
User evaluation			Y		Y
Return to work	Y		Y	Y	Y

The main outcome for this study is maintenance of physical activity, measured through self-report. Secondary outcomes are self-efficacy, perceived social support and user evaluation of the intervention. In order to link outcomes to use of the intervention we will assess perceived tailoring, and use of the intervention through web logging.

The background variables that are assessed include age, gender, education, income, alcohol use, and co-morbidity. Disease related symptoms like chest pain is assessed through WHO’s ROSE questionnaire [33]. Physical Activity is measured with the International Physical Activity Questionnaire IPAQ [34,35]. Stage of change is assessed with the scale URICA-E2 [36], which gives a more comprehensive assessment of stage than simply time before or after initiation of action. Self-efficacy is measured with modified versions of The perceived competence for regular physical exercise (PC-EX) scale [37]. Responses are given according to a scale from 0 (not at all) to 6 (to a great extent). We have previously used this scale with diabetes patients, and it performed satisfactorily with regards to both construct and predictive validity [29]. Regulatory focus is assessed with a short form of the Regulatory Focus Questionnaire [38]. This scale has been used successfully in the context of heart disease previously [39]. Social support is assessed with an adaptation of the scale from Barrera et al. [10]. Anxiety and Depression is assessed with Hospital Anxiety and Depression Scale (HADS) that is widely and successfully used for the post-discharge period that demonstrates satisfying diagnostic usefulness for screening depression symptoms and measuring anxiety of CVD patients [40]. We use the EQ-5D to measure health-related quality of life [41]. Perceived tailoring will be assessed *via* four items from Dijkstra [42]. eHealth literacy is assessed by the eHealth Literacy Scale [43].

Data on use of both interventions will be gathered through web logging. Number of log-ins, time logged in, what elements have been used most per user will be registered.

### Statistical analysis

As participants are randomized at group level, a multilevel analysis will be performed to check for clustering effects. If the intraclass correlation coefficients for our primary outcome variables are different from zero this will be taken into account in the following analyses, in order to reduce p-value bias. Potential confounders will be added as covariates. Data analysis will be performed using IBM SPSS and MLWiN. Group differences after intervention will be analysed with baseline data as a covariate in ANCOVA. Should clustering effects be found, multilevel mixed regression will be used.

## Discussion

The intervention suggested in this protocol is utilizing longitudinal tailoring based on models of health behaviour combined in a way that, to the best of our knowledge, have not been tested before. At the same time, the intervention provides the users with tools of online social interaction, but in a secure setting controlled by health professionals that commercial social interaction platforms cannot offer. In addition, the use of the randomized controlled trial methodology will help us understand if our approach succeeds in its objectives.

### Study strengths

The duration of the follow-up is one year after the discharge from the rehabilitation centre, so we will be able to investigate the long-term effects of the intervention. We are proposing the use of simple and wide spread technology in order to not challenge our users that we will be mostly middle-aged and senior citizens. The intervention has strong theoretical foundations in behavioural change models responding to the calls asking for more theory-based interventions [44], and its complexity will result in an extensively tailored system underneath the hood of a more simple user interface. The intervention is based on models of health behaviour that to a large extent have been tested independently in other eHealth interventions but – to our knowledge – it is the first time they are combined. Another strength of our study is the level of the user-involvement in the design of the intervention. As the intervention aim to fulfil the demand from the rehabilitation centre professionals and their participants, it is by definition user-initiated and we managed to maintain the high level of user-involvement by having an open dialog with them.

The study results are also expected to have high external validity, since it can be categorized as an effectiveness trial. The inclusion criteria are very broad, and in this way the results will be more relevant in the implementation of the intervention in a real-world setting, outside of strictly controlled clinical trials.

Cluster randomization will protect the blindness of the sample to the study condition. The data will be analysed in a way that will not reveal the study condition of each group, ensuring the blindness of the researchers.

The platform we are using is the Content Management System Drupal. It is an Open Source platform and this has offered many benefits to the intervention. The development costs of the study remained low, since the use of Drupal is free, making sure that funding that come from public sources was spend in the most effective and ethical way. Using an Open Source platform facilitates the reproducibility of the study with greater accuracy and at very low-cost. In addition, the continuation and the maintenance of the project don’t rely on individuals, single research institutes and software companies, but rather to a big community of contributors.

### Methodological considerations

Many of the methodological considerations of this study are related to its online nature. First of all, the population that we target, individuals with CVD history, is not in a large extent –because of their age– familiar with the Internet and the mobile technology. That makes recruitment challenging and it might introduce a bias related to the socioeconomic status of those that have access to the Internet and can take part in the study. In the same age group, we expect that women are even less familiar with new technologies and this might lead to underrepresentation of women in the sample.

### Expected outcomes, contribution and future studies

This project addresses one of the major health issues in the world, CVD, and aims to contribute to the empirical as well as theoretical basis for developing effective Internet and mobile phone based primary and secondary preventive interventions with high reach. The proposed methodology can also be applied to other health problems that can benefit from lifestyle interventions. Should the intervention prove successful, the Internet-based cardiac rehabilitation follow-up will be offered to patients at other cardiac rehabilitation facilities. It will enable the users to bring an extension of the rehabilitation services with them home, thus potentially increasing the chances of maintaining Physical Activity, which again likely will reduce rehospitalisation rates. Finally, concepts from this study can potentially be extended to primary prevention of CVD among high-risk groups.

## Trial status

The trial is open for participant recruitment and is registered in ClinicalTrials.gov (identifier NCT01223170).

## Abbreviations

ANCOVA: Analysis of covariance; CVD: Cardiovascular disease; HADS: Hospital anxiety and depression scale; HAPA: Health action process approach; IHCA: Interactive health communication applications; IPAQ: International physical activity questionnaire; PC-EX: Perceived competence for regular physical exercise; SMS: Short message service; WHO: World health organization.

## Competing interests

Both authors declare that they have no competing interests.

## Authors' contributions

KA and SCW participated in the design of the study and drafted the manuscript. All authors read and approved the final manuscript.

## Pre-publication history

The pre-publication history for this paper can be accessed here:

http://www.biomedcentral.com/1471-2261/12/50/prepub
